# Classification of myeloid neoplasms/acute leukemia: Global perspectives and the international consensus classification approach

**DOI:** 10.1002/ajh.26503

**Published:** 2022-02-25

**Authors:** Daniel A. Arber, Robert P. Hasserjian, Attilio Orazi, Vikram Mathews, Andrew W. Roberts, Charles A. Schiffer, Anne Stidsholt Roug, Mario Cazzola, Hartmut Döhner, Ayalew Tefferi

**Affiliations:** ^1^ Department of Pathology University of Chicago Chicago Illinois USA; ^2^ Department of Pathology Massachusetts General Hospital, Harvard Medical School Boston Massachusetts USA; ^3^ Department of Pathology Texas Tech University Health Sciences Center El Paso Texas USA; ^4^ Department of Hematology Christian Medical College Vellore Tamil Nadu India; ^5^ Department of Clinical Hematology Royal Melbourne Hospital and Peter MacCallum Cancer Centre Melbourne Victoria Australia; ^6^ Department of Oncology, Karmanos Cancer Institute Wayne State University Detroit Michigan USA; ^7^ Department of Clinical Medicine Aarhus University Hospital Aarhus Denmark; ^8^ Department of Molecular Medicine Fondazione IRCCS Policlinico San Matteo and University of Pavia Pavia Italy; ^9^ Department of Internal Medicine University of Ulm Ulm Germany; ^10^ Division of Hematology Mayo Clinic Rochester Minnesota USA

Since 2001, the classification of hematopoietic neoplasms has been unified under the auspices of the World Health Organization (WHO) and its affiliate International Agency for the Research on Cancer (IARC) as part of the WHO Classification series commonly referred to as “WHO Blue Books.”^1^, ^2^, ^3^ Prior to 2001, the classification of lymphomas, leukemias, and chronic myeloid disorders followed a variety of disparate and often controversial paths. For lymphoma, pathologists took the lead with limited single expert or regional classifications, such as those proposed by Rappaport,^4^ Lennert (Kiel),^5^ and Lukes and Collins^6^; an attempt to create a common language among classifications, termed the Working Formulation,^7^ effectively became its own classification. While Rappaport included leukemias and chronic myeloid disorders in his 1966 Armed Forces Institute of Pathology fascicle, accepted myeloid and leukemia classifications were largely proposed by hematologists, including the French‐American‐British Cooperative Group, the Polycythemia Vera Working Group, and others.^8^, ^9^, ^10^, ^11^, ^12^, ^13^ The criteria for these classifications varied and were based on differing combinations of clinical features, cell morphology, cytochemical studies, and in some cases, limited immunophenotyping, often with minimal, if any, evaluation of prognostic significance. Despite these limitations, the various classifications provided much‐needed criteria for the diagnoses of a variety of hematologic neoplasms, allowing for further study and refinement. None of these classifications, however, represented an international consensus or incorporated broad input from experts in hematology, oncology, genetics, and pathology.

In 1994, the International Lymphoma Study Group (ILSG), a collection of international expert lymphoma pathologists, proposed the Revised European American Lymphoma (REAL) Classification in an attempt to define biologic lymphoma entities based on a combination of clinical, morphologic, immunophenotypic, and genetic findings.^14^ Following publication of the REAL classification, Les Sobin, a co‐editor (with Paul Kleihues) of the 3rd edition Blue Book series, approached Elaine Jaffe, a co‐author of the REAL Classification and, at that time, President of the Society for Hematopathology, to develop a similar classification for the WHO/IARC Blue Book series, a series that had not previously been widely used for hematopoietic disease classification. In fact, the 2nd edition WHO series had not included hematopoietic neoplasms. Jaffe and the Society for Hematopathology Executive Committee recommended that the 3rd edition WHO effort (now to be published by IARC) be overseen by the two major hematopathology societies, the United States based Society for Hematopathology (SH) and the European Association for Haematopathology (EAHP), and that a Clinical Advisory Committee (CAC) of leading international pathologists, oncologists, hematologists, and geneticists be convened to provide input for developing such a classification. They also recommended that the 3rd edition classification not be limited to lymphoma, and should include myeloid neoplasms and acute leukemias. The first CAC was held at Arlie House, Virginia in 1997 and ultimately resulted in the 3rd edition WHO Classification of Tumors of Hematopoietic and Lymphoid Tissues in 2001, with 75 contributing authors from across the globe.^1^ Similar CACs were held in 2007 and 2014 in Chicago, hosted by James Vardiman and Michelle Le Beau, and resulted in the 4th and revised 4th edition WHO/IARC publications in 2008 and 2017, respectively.^2^, ^3^ The revised 4th edition had more than 200 contributors from 24 countries The proceedings of the CACs were all published in advance of official WHO/IARC books by the leaders of the CACs.^15^, ^16^, ^17^, ^18^, ^19^

To gain more insight into the need for clinical input on the classification of myeloid neoplasms and acute leukemia and the ramifications of such classifications on the international community, the myeloid and acute leukemia organizers of the International Consensus Conference on the Classification of Myeloid and Lymphoid Neoplasms asked for additional perspectives from leaders from four continents.

## Perspective from Professor Andrew Roberts, Theme Leader, Cancer Research and Treatments, The Walter and Eliza Hall Institute of Medical Research, Metcalf Chair of Leukemia Research, University of Melbourne, Clinical Hematologist, Clinical Hematology, Royal Melbourne Hospital & Peter MacCallum Cancer Centre, Australia

The care of patients with newly diagnosed hematological malignancies has never been in greater flux. With greater power than ever before to probe differences and commonalities in genetic abnormalities between patients, and equipped with an increasing armamentarium of targeted therapies, each with their own more selective range of activity, physicians, and patients are now faced with more decisions than ever. These advances bring the promise of precision medicine several steps closer to reality, but inevitably generate greater uncertainty. For physicians, this uncertainty becomes apparent as we ask ourselves important questions, such as: how sure am I that this is the right diagnosis for this condition; have I ordered all the necessary tests and properly interpreted them; this test result does not seem to fit the diagnosis, is that a problem; does this diagnosis dictate what is the best treatment; and will that same treatment work if I have not made the correct diagnosis? For patients receiving transparent information and advice from their physician, this uncertainty can be a barrier to accepting considered recommendations or increase susceptibility to the negative effects of misinformation spread via social media.

Central to enabling rational and evidence‐backed decision making in clinical practice is getting the diagnosis correct. Hematology has advanced because our forebears have assimilated insights to the pathophysiology of disease into the clinical and morphological algorithms that generate our current integrated diagnostic classification system. As our knowledge grows, our diagnostic classification system must evolve. In this light, it is time for the classification of myeloid neoplasms and acute leukemia to be updated. We need it to further integrate robust patterns of morphology, immunophenotype, cytogenetics, and molecular aberrations into rational diagnostic categories. In doing so, we need to be continually mindful of the performance characteristics of new molecular tests. However, classification systems should lead hematology forward, and not be held back by variable issues in test implementation. The latter will be most rapidly solved when their importance to diagnosis is considered established and demanded by physicians and patients alike.

In Australia, a consortium of professional and consumer non‐government organizations has recently assessed the performance of our health system in caring for people with hematological malignancies.^20^ Despite excellent outcomes for many patients, gaps have been identified where outcomes can be improved by applying current knowledge. Loud among the calls are the voices advocating equity of access for all patients to receive an accurate diagnosis. Patients know that an accurate diagnosis flows understanding of their prognosis and access to best available therapy. They expect medicine to be able to evaluate their disease with appropriate and precise tests and to categorize it with confidence (i.e. with as little uncertainty as possible). The evolving classification systems for hematological malignancies have served us well in that respect. Looking forward to the next iteration of international classification, I urge all stakeholders—pathologists, physicians, manufacturers, and payers—to work together to enable its implementation so the benefits to patients fully flow.

## Perspective from Dr Vikram Mathews, Professor of Hematology, Christian Medical College, Vellore, India

While it is common parlance to state that “recent advances in our understanding of the biology of myeloid neoplasms has been transformative,” for the most part, this is relevant to a fifth of the world's population that lives in the developed world.^21^ Myeloid neoplasms such as AML are the most expensive diseases to treat, complicated further in developing countries by high out‐of‐pocket expenses, susceptibility to fungal and multi‐drug resistance bacterial infections, and limited access to centers of excellence.^22^ Additionally, recently approved blockbuster drugs for myeloid neoplasms are, for the most part, not available or are accessible to a small group of affording patients. An exception is the treatment of chronic myeloid leukemia (CML), where the use of generic tyrosine kinase inhibitors (TKIs) has genuinely transformed the therapeutic outcome of patients in these countries, despite repeated and concerted efforts to discredit them.^23^ Data purported to be of clinical relevance with high‐level evidence (Phase III RCT) generated in a clinical trial setting from a highly developed country with universal health care access in AML will almost certainly not be broadly applicable in India. It is also important to recognize that in India it has been estimated that approximately 39 million people fall below the poverty line every year due to the high out‐of‐pocket expenses associated with health care access.^24^, ^25^

The relevance of many of the above observations on the classification of malignancies in general and, more specifically, the classification of myeloid malignancies in low and upper‐middle‐income countries (L/UMIC) can be considered under the following main headings: (1) while molecular classification and the anticipated spinoffs of better‐targeted therapies with minimal off‐target side effects must be encouraged it is also essential to continue to retain the relevance of the basic tools of classification such as morphology, cytochemistry, and immunophenotyping, which for the most part, when done well, is adequate for appropriate therapeutic decision making. (2) Rare entities need to be clearly stated as being rare. There is a tendency for excessive attention to the rare and exotic at the expense of common and curable malignancies. Such attention is exacerbated in an environment where such knowledge rather than actual practice is disproportionately valued and rewarded, a self‐fulfilling prophecy dictated by the existing circumstances in L/UMIC. (3) An effort needs to be made to highlight highly curable/easily managed myeloid neoplasms and integrate into the classification the optimal standard of care of such conditions. (4) Similarly, strategies for rapid and accurate diagnosis of very poor prognostic markers must be highlighted, where possible, this is very relevant in L/UMIC where such data would quickly change the decision to best supportive care rather than proceeding with expensive therapies that could potentially drive a patient's family to destitute poverty. (5) A more significant effort is required with prognostication and prediction of clinical outcomes by including, where possible, data from L/UMIC to make the classification more relevant to 80% of the world's population.

## Perspective from Dr Anne Stidsholt Roug, Consultant and Associate Professor, Aarhus University Hospital, Denmark

In Europe, over the past decades, much effort (and generous funding) has been allotted into molecular testing both at the community level and in clinical trials. It is recognized that molecular phenotype holds prognostic information and is increasingly being used as a decision‐making tool in terms of both targeted drug therapies and allogeneic hematopoietic stem cell transplant. Accordingly, many centers in Europe have embraced extensive molecular profiling as part of the routine work up of patients with acute leukemia eligible for intensive chemotherapy, and this approach is also increasingly being applied in elderly or frail patients; however, access to such analyses in Europe remains inequitable and the platforms not well standardized.

After more than 10 years of molecular profiling of myeloid neoplasms and acute leukemia, it can be argued that the list of drugs inspired by genomics remains relatively small and the pipelines for the development of other such targeted therapies limited. Also, in diagnostics and risk stratification, data from molecular profiling are yet to be readily translated into non‐overlapping disease entities. Furthermore, molecular association of diseases is often confounded by age and classification into low‐ versus high‐risk categories does not always hold true. In some European countries, national and government‐funded initiatives for genome‐wide sequencing are being established. However, most data derived from molecular testing may be redundant when applied to current classification and risk stratification systems.

In the (2016) WHO classification of Myeloid Neoplasms and Acute Leukemia, eight subtypes of AML are defined by specific cytogenetic abnormalities and certain mutations.^18^ A similar approach was undertaken by The European LeukaemiaNet (ELN) initiative, to risk‐stratify AML, based on 12 cytogenetic and 6 mutational aberrations.^26^ However, both of these classification and risk stratification systems represent oversimplification of a much more complicated underlying biology, thus warranting the need for continuous elaboration of genetically driven diagnostics, in the context of developing knowledge bases ensuring harmonized interpretation of genetic findings, use of artificial intelligence and automation for developing decision support tools integrating clinical and genetic data for risk scoring systems as well as solid studies combining clinical and molecular data for optimizing and personalizing treatment of patients. With these issues in mind, classification of hematological neoplasms into stringently defined disease entities of clinical relevance and biological homogeneity requires further amelioration and integration of all today's diagnostic tools continuously empowered by new molecular information. In Europe, collective efforts such as the ELN, including 220 participating centers in 44 countries, has provided high‐impact research papers, guidelines, and updated management recommendations and represents a cooperative that has formally started this work.

## Perspective from Dr Charles A. Schiffer, Emeritus Professor of Oncology, Karmanos Cancer Institute, Wayne State University School of Medicine, USA

The International Consensus Classification (ICC) for hematologic malignancies provides an important update from previous classification systems by incorporating newer molecular and clinical information, adding newer entities, and clarifying diagnoses which were labeled “provisional” in earlier endeavors. Expert subspecialty hematopathologists and clinicians cooperated in this effort, despite the pandemic, using a combination of virtual meetings (less fun) and an actual face‐to‐face meeting made possible by the protective benefit of more widespread vaccination.

Why bother? Aside from calling clinical and diagnostic laboratory attention to the handful of diagnoses with unique mutations which benefit from truly targeted therapies, standardization of diagnosis is critical to assure that patient populations within and across clinical trials are comparable, so that results using different therapies can be compared. Parallel efforts standardizing definitions of response and toxicity from treatment also provide assurance that we are all talking the same language. Although more precise pathologic diagnosis often provides guidance about treatments, there is usually little additional insight provided about the pathophysiologic mechanisms governing treatment choice. For example, why are some morphological subtypes more s*ensitive* to treatment with a high probability of cure? Think CHOP for diffuse large B cell lymphoma and its kissing cousin follicular lymphoma—significant cure rate for the former, great responses but low cure rate for the latter. Why the exquisite sensitivity of hairy cell leukemia to purine analogs with much less activity against other lymphoproliferative cancers at similar stages of differentiation? My group published one of the earlier large studies of cytogenetics in AML, noting as did everybody else, that patients with CBF AML (t(8;21) and inv16) had appreciable cure rates with standard anthracycline and cytarabine regimens.^27^ I naively expected that the explanation might be rapidly forthcoming, but I am now much older, and still await clues potentially applicable to other leukemias. Similarly, in all, why does extreme hyperdiploidy confer sensitivity when we usually associate aneuploidy with a more malignant phenotype? There are many other such examples which could be cited.

Acknowledging that it is experimentally more straightforward to study resistance than sensitivity, there are major biologic lessons to be learned from hematologic cancers that we cure. Given the heterogeneity of the curable hematologic malignancies, it would seem unlikely that extreme chemosensitivity producing cures is simply a consequence of the absence of resistance mechanisms. It is attractive to hypothesize that recovery of immune surveillance plays a role, with reversal of the immune tolerance which was permissive of the growth of the initial transformed cells, since this might be amenable to pharmacologic manipulation. A classification system lumping the heterogeneous chemotherapy curable cancers together with the goal of identifying biologic similarities would be of interest and could be the next scientific focus of groups such as the ICC.

## The International Consensus Classification

The perspectives above underscore the highly impactful nature of changes in the classification of myeloid neoplasms and acute leukemia, and how such changes clearly necessitate a broad CAC effort prior to any new classification. The organizers of the International Consensus Conference agreed and felt it to be essential to clarify to the hematology and pathology community the steps that led to this process.

Despite several exchanges between leaders of SH and EAHP and clinical leaders with Ian Cree, the WHO/IARC 5th edition Blue Book series editor, including a formal request from 33 leaders in pathology, hematology, oncology, and genetics in February 2020, no consensus was reached on the need to convene a clinical advisory committee (CAC) prior to initiating the 5th edition classification. Accordingly, the decision was made to move ahead with a CAC, knowing that it may not be possible for its conclusions to inform the upcoming WHO classification. This decision was not made lightly and involved input from the prior WHO editors and senior advisors as well as leadership of SH and EAHP and leading clinicians from across the globe. To organize this International Consensus Conference (ICC) on the Classification of Myeloid and Lymphoid Neoplasms, clinical and pathology co‐chairs for separate lymphoma and myeloid/acute leukemia sections were selected. The myeloid/acute leukemia pathology co‐chairs, Daniel Arber, Robert Hasserjian, and Attilio Orazi, and the clinical co‐chairs, Mario Cazzola, Hartmut Döhner, and Ayalew Tefferi, proposed participants with a goal to include diverse and geographically broad leaders in the various disease categories. Once participants were identified, a tremendous amount of advance work was necessary to prepare for a productive two‐day CAC meeting. Invited participants were assigned to subgroups, which each reviewed in advance the existing classification and proposed potential changes and key questions to be addressed at the CAC. Each subgroup held a series of virtual meetings and key issues that crossed disease groups, such as potential blast cell count changes or exclusion criteria, were shared between groups.

The efforts of the subgroups came together as presentations to the entire CAC on September 20–21 at the Rubenstein Forum on the campus of the University of Chicago. The combined lymphoma and myeloid/acute leukemia CAC included 138 participants (42 in person and 98 remote) from 23 countries and 5 continents. Potential classification changes were discussed and debated among the participants, with new questions and unresolved issues identified. Following the meeting, the subgroups reconvened to resolve key issues and to propose a final classification of myeloid neoplasms and acute leukemias. For several controversial issues, the entire CAC myeloid/acute leukemia participants were polled to obtain further input and ensure a consensus on important changes. The results will be the International Consensus Classification of Myeloid and Lymphoid Neoplasms (ICC‐MLN), which will be reported in peer‐reviewed manuscripts with a publication aim of mid‐2022. The new name was proposed by the pathology co‐chairs and reflects the broad, international input from expert pathologists and clinicians with updated genetic integration. The ICC‐MLN will reflect a consensus on disease entities, terminology, and diagnostic criteria between all parties involved in the diagnosis and treatment of patients with hematologic neoplasms.

The basic tenet of the new ICC classification is reliance on expert pathology and clinical input in crafting a genetically integrated and clinicopathologically sound classification.

## Concluding remarks

The Clinical Advisory Committee process is unique to the classification of hematopoietic neoplasms, especially for the myeloid neoplasms and acute leukemias. A usable disease classification is more than a list of disease names proposed by a single person or small group of individuals, irrespective of their expertise. Establishing a consensus on the detailed criteria that define disease categories is essential for pathologists to be able to make diagnoses that are reproducible around the world. Without well‐established criteria, diagnostic ambiguity occurs which impacts treatment and outcome of individual patients and the ability of clinical trials to provide meaningful results. For this reason, we consider it essential to have convened a CAC of international experts in 2021 in order to revise the existing classification of myeloid neoplasms and acute leukemias that will be used to guide patient management.

## CONFLICT OF INTERESTS

The authors declare that there are no conflict of interests.

## AUTHOR CONTRIBUTIONS

All authors contributed to writing this manuscript.

## Data Availability

Data sharing is not applicable to this article as no new data were created or analyzed in this study.
